# Prevalence and Predictors of Overweight and Obesity Among Kenyan Women

**DOI:** 10.5888/pcd15.170401

**Published:** 2018-04-19

**Authors:** Rahma S. Mkuu, Katrina Epnere, Muhammad Abdul Baker Chowdhury

**Affiliations:** 1 Department of Health and Kinesiology, Texas A&M University, College Station, Texas; 2 Statistics Collaborative, Inc, Washington, DC; 3 Department of Emergency Medicine, University of Florida College of Medicine, Gainesville, Florida

## Abstract

**Introduction:**

Overweight and obesity are associated with increased rates of chronic disease and death globally. In Kenya, the prevalence of overweight and obesity among women is high and may be growing. This study aimed to determine the national prevalence and predictors of overweight and obesity among women in Kenya.

**Methods:**

We used cross-sectional data from the 2014 Kenya Demographic and Health Survey (KDHS). Data on body mass index for 13,048 women (aged 15–49 y) were analyzed by using multivariable logistic regression models. Overweight and obesity were classified by using World Health Organization categories (normal weight, 18.5 to <24.9; overweight, 25.0 to <29.9; and obese, ≥30.0).

**Results:**

The prevalence of overweight was 20.5%, and the prevalence of obesity, 9.1%. Women aged 35 to 44 (odds ratio [OR] = 3.14; 95% confidence interval [CI], 2.58−3.81), with more than a secondary education (OR = 1.43; 95% CI, 1.05–1.95), married or living with a partner (OR = 1.73; 95% CI, 1.42−2.08), not working (OR = 1.27; 95% CI, 1.10–1.48), in the richest category (OR = 6.50; 95% CI, 5.08–8.30), and who used hormonal contraception (OR = 1.24; 95% CI, 1.07–1.43) were significantly more likely to be overweight or obese.

**Conclusion:**

A high proportion of women in Kenya are overweight or obese. Our study indicates that women from urban areas and women with high socioeconomic status make up the largest proportion of women who are overweight or obese. Targeted and tailored studies and interventions are needed to identify evidence-based obesity prevention strategies for high-risk women in Kenya.

## Introduction

Globally, the prevalence of overweight and obesity has more than doubled since 1980 (1). In 2016, more than 1.9 billion adults were either overweight or obese; of whom more than 650 million were obese (1). The rate of overweight and obesity is higher among women than among men worldwide (1). Overweight and obesity is associated with a higher prevalence of major cancers, diabetes, and cardiovascular diseases (2–5). Additionally, overweight or obese women are at substantially higher risk of negative maternal outcomes such as gestational diabetes, pre-eclampsia, induction of labor, cesarean sections, and postpartum hemorrhage (3,6).

Overweight and obesity affects more than the health of the woman; it also affects the health outcomes of offspring. For pregnant mothers, overweight and obesity increases risk for neonatal death and malformations and the delivery of low-birth-weight infants (3,6). Children born of mothers who are overweight or obese, compared with children born of mothers who are not overweight or obese, are less likely to be breastfed, are breastfed for shorter lengths of time, have a higher risk of chronic conditions, and are more likely to be overweight or obese (3,7). Furthermore, undernutrition during childhood is also associated with increased obesity risk; as a result, some countries have a double burden of undernutrition and overweight and obesity (1,8).

Rates of overweight and obesity among women are increasing in low-income and middle-income countries (LMICs) (4,9–10). A growing body of literature demonstrates the increasing prevalence of overweight and obesity among women in sub-Saharan Africa (11,12). This increasing prevalence is associated with urbanization, access to and consumption of high-calorie diets, and decreased physical activity (12–14). Women in sub-Saharan Africa are also faced with adverse maternal and child health outcomes; therefore, overweight and obesity may aggravate maternal and child health challenges (15,16).

In Kenya, regional studies reported a higher prevalence of overweight and obesity among women than among men; in one study, 43.4% of women and 34% of men were overweight or obese (17–20). Overweight and obesity in Kenya increases the risk for raised blood glucose levels (20) and high blood pressure (20,21).

Similar to predictors in other LMICs, predictors for overweight and obesity in Kenya include living in urban areas, high income, and high levels of education (4,5,8,14,17,18,22,23). Studies conducted among slum dwellers also provide evidence of high rates of overweight and obesity among low-income groups (19). One explanation for higher rates of overweight and obesity among high-income groups is consumption behaviors. In urban Kenya, one study found that high-income women had a higher prevalence of overweight and obesity and were more likely to consume high-caloric, high-fat, and high-protein foods associated with higher risk of overweight and obesity (17). Other predictors among women include increased age, increased parity, being divorced or widowed, higher alcohol intake, insufficient intake of fruits and vegetables, and decreased physical activity (8,23).

Despite emerging literature on the scope of overweight and obesity in Kenya, a gap exists in understanding nationwide predictors of overweight and obesity among women. Studies are mainly regional (18,23) or among selected groups such as slum dwellers (19,20). The objective of our study was to estimate the prevalence and explore predictors of overweight and obesity among Kenyan women by using data from the most recent (2014) administration of the Kenya Demographic and Health Survey (KDHS).

## Methods

This study used secondary data from the 2014 KDHS. The 2014 KDHS was reviewed, approved, and conducted by the ICF Macro Institutional Review Board and the ethical board of the Kenya National Bureau of Statistics. The 2014 KDHS complies with all requirements of the US Department of Health and Human Services’ Title 45 Code of Federal Regulations Part 46, Protection of Human Subjects (24). For each participant, verbal consent was obtained before the interview and data collection (25). Details about the sampling, survey design, survey instruments, and quality control are described elsewhere (25).

### Sampling strategy and sample size

The 2014 KDHS used 2-stage stratified random sampling. First, 1,612 enumeration areas were selected with equal probability from the fifth National Sample Survey and Evaluation Programme sampling frame. Second, a sample of 25 households was selected from each cluster to provide statistically reliable estimates of key demographic and health variables for the country as a whole. The sampling methods of the 2014 KDHS resulted in a total sample size of 40,300 households. To address concerns of data quality and management of the study due to sample magnitude, a decision was made not to conduct the entire questionnaire in every household. Instead, a shorter questionnaire was constructed by using priority indicators that were identified by key stakeholders at the county level. As a result, some households received the entire questionnaire (hereinafter referred to as the long questionnaire) and some received the short questionnaire. The 2014 KDHS identified 15,317 women as eligible to participate in the survey; of these women, 14,741 were interviewed (response rate, 96%). One in every 2 households was selected to receive the long questionnaire.

We used data only from the women’s long questionnaire; these files included women aged 15 to 49 years. We then selected women whose height and weight information was available to calculate body mass index (BMI). Of the 14,741 women who were interviewed, we excluded 319 for whom information on height and/or weight were missing (25). We also excluded 967 women who were pregnant at the time of the survey and 407 women who were in their postnatal period. The final sample size was 13,048. 

BMI was considered the primary outcome of interest (dependent variable). BMI is used as an indicator for body fat and is defined as weight in kilograms divided by height in meters squared (kg/m^2^). For this study, we used the World Health Organization’s definition. Participants with a BMI of less than 25.0 were not considered to be overweight or obese, whereas participants with a BMI of 25.0 or more were categorized as overweight or obese.

We considered the following to be predictors of overweight or obesity: age in years (categorized as 15–24, 25–34, 35–44, and 45–49), region (Coast, North Eastern, Eastern, Central, Rift Valley, Western, Nyanza, or Nairobi), type of residence (rural or urban), wealth index (poorest, poorer, middle, richer, or richest), educational level (no education, primary, secondary, or higher than secondary), working status (yes or no), marital status (never in union, in a union [married or living with a partner], or not in a union [widowed, divorced, or separated]), status of contraceptive use (not using any method; using hormonal methods [pill, intrauterine device, injection, implant]; other methods [female/male condom, female/male sterilization, withdrawal], physical activity (yes or no), and alcohol consumption (yes or no) (4,5,14,17,18,20,22,23,26–28). The KDHS estimated a household wealth index by using multiple household and asset variables; validated principle component analysis was used for the estimation (29). The principal component analysis took into consideration individual households on a continuous scale of relative wealth, named a wealth index score. Then wealth quintiles were determined by assigning the wealth index score to each household member, ranking each person by his or her score, and then dividing the ranking into 5 equal categories, each comprising 20% of the population. 

We first conducted a descriptive analysis of the study sample. We then performed bivariate analysis (χ^2^ test) to test the association of selected categorical variables with the dependent variable. A *P* value of  < .20 was chosen arbitrarily as the criterion for including variables in the multivariable logistic regression model, and results were considered significant at *P*  ≤  .05. Using survey logistic regression procedures, we estimated the odds ratios (ORs) and 95% confidence intervals (CIs) for each covariate to identify predictors of overweight or obesity. The sample stratification and clustering effect were used to apply survey weights to individual responses in the sample to reflect the age, sex, and geographical distribution of the Kenyan population. We used forward, backward, and stepwise model selection procedures to select the best predicting model. Before entering the independent variables into the multivariable models, we checked the variation inflation factor to avoid the problem of multicollinearity. To assess the overall fit of the final model, we used the Pearson χ^2^ test and Hosmer–Lemeshow goodness-of-fit test. We managed all data in SAS version 9.4 for Windows (SAS Institute Inc) and performed all statistical tests and modeling exercises in Stata/MP 13 (StataCorp LLC).

## Results

We found that 20.5% of women in Kenya were overweight and 9.1% were obese; overall, approximately one in 3 (32.8%) women in Kenya were either overweight or obese. We found a significant association between being overweight or obese by age, region, type of residence, education, wealth index, marital status, alcohol consumption, and working status (Table 1). Almost 45% of study participants aged 35 to 44 and 48.4% of participants in the group aged 45 to 49 were overweight or obese. By region, the highest prevalence of overweight and obesity was in Nairobi (47.8%), followed by the Central (47.0%) and Coast (32.4%) regions. Overweight and obesity was more prevalent among urban residents (43.5%) than among rural residents (26.0%). More than 35% of those with a secondary education and 45.7% of those with more than a secondary education were overweight or obese. By wealth index, overweight and obesity was more prevalent among the richer (41.1%) and richest (50.1%) groups. The prevalence of overweight and obesity was higher (39.7%) among women who were married or living with a partner than among women who were widowed, divorced, or separated (38.2%) or who were never in a union (18.4%). Overall, 62.0% of all participants reported that they were currently working; 39.5% of working participants were overweight. The difference in prevalence of overweight or obesity by physical activity status (32.6% active vs 33.2% not active) and by alcohol consumption (33.0% among alcohol consumers vs 32.5% among nonconsumers) was not significant. By contraceptive use, we found the highest prevalence of overweight and obesity among those who used hormonal contraceptives (41.5%). Among women from urban areas, 28.6% were overweight and 14.9% were obese, whereas among women from rural areas, 18.9% were overweight and 7.1% were obese (Figure).

**Table 1:** Socioeconomic, Demographic, and Anthropometric Characteristics of Study Participants (N = 13,048), by Weight Status, Kenya Demographic and Health Survey, 2014

Variable	Not Overweight, No. (%)	Overweight or Obese, No. (%)	*P* Value^a^
**Age group, y**			
15–24	3,909 (83.3)	786 (16.7)	<.001
25–34	2,694 (61.0)	1,724 (39.0)	
35–44	1,577 (55.2)	1,280 (44.8)	
45–49	526 (51.6)	494 (48.4)	
**Region**			
Coast	845 (67.6)	401 (32.4)	<.001
North Eastern	190 (81.2)	44 (18.8)	
Eastern	1,325 (69.8)	575 (30.3)	
Central	893 (53.0)	792 (47.0)	
Rift Valley	2,359 (71.6)	949 (28.7)	
Western	1,062 (75.5)	345 (24.5)	
Nyanza	1,426 (73.3)	455 (26.7)	
Nairobi	786 (52.2)	719 (47.8)	
**Type of residence**			
Urban	2,938 (56.5)	2,261 (43.5)	<.001
Rural	5,767 (74.0)	2,022 (26.0)	
**Education**			
No education	689 (82.2)	150 (17.9)	<.001
Primary	4,529 (69.4)	2,001 (30.6)	
Secondary	2,734 (64.6)	1,500 (35.4)	
Higher	753 (54.3)	633 (45.7)	
**Wealth index**			
Poorest	1,668 (87.7)	233 (12.3)	<.001
Poorer	1,831 (78.9)	491 (21.1)	
Middle	1,868 (72.4)	711 (27.6)	
Richer	1,647 (58.9)	1,149 (41.1)	
Richest	1,692 (49.9)	1,700 (50.1)	
**Marital status**			
Never in union	3,236 (81.6)	727 (18.4)	<.001
In union (married or living with partner)	4,516 (60.4)	2,967 (39.7)	
Not in a union (widowed, divorced, or separated)	953 (61.8)	598 (38.2)	
**Drinks alcohol**			
No	8,338 (67.5)	4,014 (32.5)	.004
Yes	368 (57.8)	268 (33.0)	
**Currently works**			
No	3,837 (77.6)	1,106 (22.4)	<.001
Yes	4,857 (60.5)	3,174 (39.5)	
**Physical activity**			
No	5,235 (66.8)	2,602 (33.2)	.69
Yes	3,468 (67.4)	1,680 (32.6)	
**Contraceptive use**			
Not using any method	5,074 (74.2)	1,766 (25.8)	<.001
Hormonal method	2,806 (58.5)	1,992 (41.5)	
Other method	825 (61.1)	525 (38.9)	

a Determined by χ^2^ test; *P* values ≤ .05 considered significant.

**Figure:**
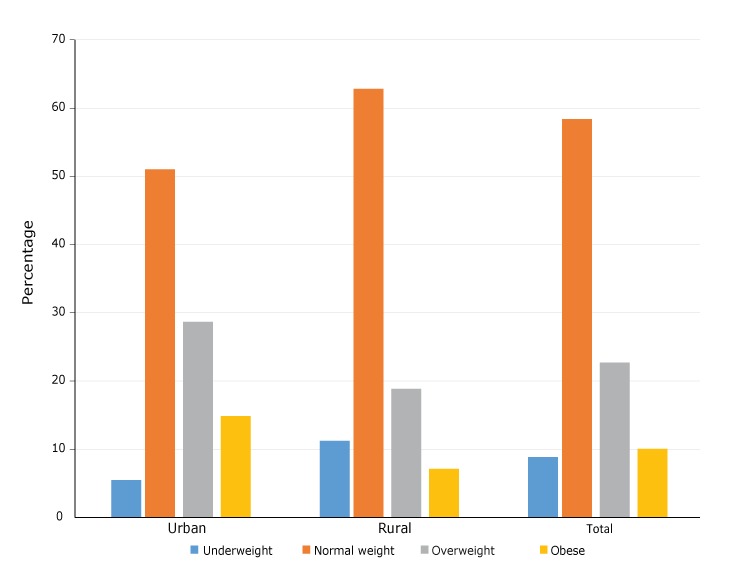
Prevalence of overweight and obesity among rural and urban women of Kenya. Data source: 2014 Kenya Demographic and Health Survey. **Table:** 

Weight class	Urban, %	Rural, %	Total, %
Underweight	5.5	11.2	8.8
Normal weight	51.0	62.8	58.4
Overweight	28.6	18.9	22.7
Obese	14.9	7.1	10.1

All significant covariates from the multivariable logistic regression model are presented in Table 2. Odds of being overweight or obese were significantly associated with older age, type of residence, education level, wealth index, marital status, working status, and contraceptive use. The risk of being overweight or obese was significantly higher among all 3 age groups compared with women aged 15 to 24. Women in rural areas were 17% (adjusted OR = 0.83; 95% CI, 0.72–0.97) less likely to be overweight or obese than were women in urban areas.

**Table 2:** Odds Ratios and 95% Confidence Intervals of Correlates of Overweight and Obesity, Kenya Demographic and Health Survey, 2014

Variable	Odds Ratio (95% Confidence Interval)	*P* Value^a^
**Age group, y**		
15–24	1 [Reference]	
25–34	2.08 (1.74–2.49)	<.001
35–44	3.14 (2.58–3.81)	<.001
45–49	4.13 (3.26–5.24)	<.001
**Type of residence **		
Urban	1 [Reference]	
Rural	0.83 (0.72–0.97)	.02
**Education level**		
No education	1 [Reference]	
Primary	1.33 (1.03–1.73)	.03
Secondary	1.54 (1.18–2.02)	.002
Higher	1.43 (1.05–1.95)	.02
**Wealth index**		
Poorest	1 [Reference]	
Poorer	1.77 (1.42–2.00)	<.001
Middle	2.50 (2.02–3.09)	<.001
Richer	4.37 (3.51–5.46)	<.001
Richest	6.50 (5.08–8.30)	<.001
**Marital status **		
Never in union	1 [Reference]	
In union (married or living with partner)	1.73 (1.42–2.08)	<.001
Not in a union (widowed, separated, or divorced)	1.54 (1.22–1.95)	<.001
**Currently works **		
No	1.27 (1.10–1.48)	.001
Yes	1 [Reference]	
**Contraceptive use **		
Not using any method	1 [Reference]	
Hormonal method	1.24 (1.07–1.43)	.004
Other method	1.08 (0.88–1.33)	.46

a Determined by logistic regression.

Compared with women with no education, women with a secondary education were 54% (OR = 1.54; 95% CI, 1.18–2.02) more likely and women with more than a secondary education were 43% (OR = 1.43; 95% CI, 1.05–1.95) more likely to be overweight or obese. Richer women (OR = 4.37; 95% CI, 3.51–5.46) and the richest women (OR = 6.50; 95% CI, 5.08–8.30) were more likely to be overweight or obese than were the poorest women. The risk of being overweight or obese was 73% higher among women who were married or living with a partner (OR = 1.73; 95% CI, 1.42–2.08) than among women who were never in a union. Nonworking women had 27% higher risk (OR = 1.27; 95% CI, 1.10–1.48) of being overweight or obese than had women who were working. Women who used hormonal contraceptives (OR = 1.24; 95% CI, 1.07–1.43) had a higher risk of being overweight or obese compared with women who did not use any contraceptive method. The Hosmer–Lemeshow goodness-of-fit test for the model comparing overweight or obese and not overweight or obese produced an *F* value of 1.73 (*P* = .08). 

## Discussion

The primary objective of this study was to estimate the prevalence and examine predictors of overweight and obesity among women in Kenya by using a nationally representative sample. We found that 20.5% of women in Kenya were overweight and 9.1% were obese; overall, approximately one in 3 (32.8%) women in Kenya are either overweight or obese. Residing in an urban area, having a high level of education or wealth, being in a union, using hormonal contraception, and not working were significant predictors of increased odds of being overweight or obese. To the best of our knowledge, this is the first nationally representative study to examine the prevalence and predictors of overweight and obesity among women in Kenya.

Previous studies conducted mainly in community settings also demonstrated high rates of overweight and obesity among women in Kenya; these studies reported prevalence as high as 43% (17,19). Other LMICs found similar prevalence rates among women (12). A study of participants in the Demographic and Health Survey in 1998 in South Africa found that 56.6% of women were overweight or obese (12).

Our findings match the findings of previous studies that associated overweight and obesity with older age (17,19,23). Women aged of 40 to 44 had more than 4 times the odds of being overweight or obese than women aged 15 to 24 in our study. The trend of increasing BMI by age is concerning because older age is also associated with increased risk for noncommunicable chronic diseases in Kenya (20,23). Interventions that target the modifiable risk factor of overweight and obesity can help to curb the increasing burden of noncommunicable disease in Kenya.

Although overweight and obesity was not significantly associated with participants’ region of residence, those from Nairobi and Central region had a high rate of overweight and obesity, 47% or more. Research on obesity in Kenya is dominated by studies conducted in Nairobi region (19–21,23); our findings suggest a need for more regionally representative studies. A significant predictor was type of residence: respondents in urban areas had higher odds of being overweight or obese than respondents in rural areas. This finding is supported by several other studies in developing countries and in Kenya that demonstrate higher rates of overweight and obesity among urban dwellers (5,17,30). In Kenya, one study found the prevalence of obesity to be 60.3% among urban residents and 19.5% among rural residents (18). The higher risk of obesity among women in urban areas of Kenya is associated with increased consumption of high-calorie, high-fat diets (17). To the best of our knowledge, no interventions to curb overweight and obesity among Kenyan women have been described in the scientific literature. Evidence-based interventions aimed at preventing and reducing overweight and obesity, especially among urban women in Kenya, are needed.

As other studies in LMICs have found, our study found increased risk of overweight and obese among women from higher socioeconomic groups (17,18). Having obtained a secondary education or more than a secondary education was associated with increased obesity. Additionally, women in the highest wealth categories had more than 6 times the odds of being overweight or obese than women in poorest category. Other studies found that wealthy women in Kenya have an increased risk for obesity (18,23). This increased risk among women of high socioeconomic status is associated with greater consumption of high-calorie, high-fat diets (17). To our surprise, nonworking women had higher odds than working women of being overweight and obese; this might be explained by the fact that women in a union were significantly more likely to be overweight and obese than women never in a union. Women might have a higher risk of being overweight or obese because of greater income and because they have more children than their never-married counterparts (13).

Our study also found that women who used hormonal contraception (pills, intrauterine device, injections, or implants) were more likely to be overweight or obese than women not using any method. Our findings are supported by research demonstrating an increase in weight as a result of hormonal contraception (26). However, some studies refute our findings and report no significant differences in weight gain as a result of contraceptive use (27). The results of our study should be interpreted with caution. A systematic review examining the relationship between contraceptive use and weight gain found mixed and limited evidence of weight gain due to contraceptive use (31). Additionally, obese women gained more weight than their nonobese counterparts when they initiated contraception (7). Thus, the relationship between weight gain and contraception use might be more complex than we realize and calls for more rigorous examination. 

Our study has several limitations. First, because KDHS collects cross-sectional data, our analysis could not explore changes over time. Second, data on several important variables, such as women’s food-consumption behaviors, were not collected by KDHS, and KDHS did not objectively measure physical activity; therefore, our ability to examine the association of these variables with overweight and obesity was restricted. Third, numerous psychological factors (eg, depressive and anxiety disorders) and physiological factors may also be associated with obesity, but these factors were not included in this study because they were not available in the data set. Finally, because the data were available only for women aged 15 to 49, our results may not be generalizable to girls younger than 15 and women older than 49.

Despite these limitations, our results contribute to the literature on the prevalence and association between sociodemographic variables and the status of overweight and obesity among women in Kenya. The major strength of this study is that it is the first to use nationally representative data to examine the prevalence and predictors of overweight and obesity in this country. The 2014 KDHS is the first nationally representative sample to collect BMI data. The use of these data to examine overweight and obesity is critical and allows for timely analysis. Second, because the KDHS uses standard and validated data-collection tools, the measurement error and bias are smaller in our study than in other small-scale cross-sectional studies in Kenya.

Our study found a high prevalence of overweight and obesity among Kenyan women in 2014. This nationally representative sample resulted in demonstrating that older women, urban dwellers, women with a high socioeconomic status, women taking hormonal contraception, and married women had higher odds of being overweight or obese. Given the growing prevalence of overweight and obesity globally, there is a need for interventions aimed at reducing overweight or obesity among Kenyan women. Interventions in this country should consider taking a multisectoral approach to address the sociostructural determinants of health, such as obesogenic urban environmental factors, that influence overweight and obesity. Curbing overweight and obesity among Kenyan women is likely to reduce the burden of chronic noncommunicable diseases in this country.
